# Repair of Abdominal Wall Defects *In Vitro* and *In Vivo* Using VEGF Sustained-Release Multi-Walled Carbon Nanotubes (MWNT) Composite Scaffolds

**DOI:** 10.1371/journal.pone.0064358

**Published:** 2013-05-22

**Authors:** Zhicheng Song, Zhi Yang, Jianjun Yang, Zhengni Liu, Zhiyou Peng, Rui Tang, Yan Gu

**Affiliations:** Department of General Surgery, Shanghai Ninth People's Hospital Affiliated to Shanghai JiaoTong University School of Medicine, Hernia and Abdominal Wall Surgery Center of Shanghai Jiao Tong University, Shanghai, China; National University of Ireland, Galway, Ireland

## Abstract

**Objective:**

Porcine acellular dermal matrices (ADM) have been widely used in experimental and clinical research for abdominal wall repair. Compared to porcine small intestinal submucosa (SIS), the effect of these matrices on the regenerative capacity of blood vessels is still not ideal. Multi-walled carbon nanotubes (MWNTs) can more effectively transport VEGF to cells or tissues because of their large specific surface area and interior cavity. In this study, we explored the safety and efficacy of implanted VEGF-loaded MWNT composite scaffolds *in vitro and vivo* to repair abdominal wall defects.

**Materials and Methods:**

VEGF-loaded MWNTs were prepared by a modified plasma polymerization treatment. Four composite scaffolds were evaluated for cytotoxicity, proliferation, and release dynamics. We created 3 cm×4 cm abdominal wall defects in 43 Sprague-Dawley rats. After implantation times of 2, 4, 8, and 12 weeks, the scaffolds and the surrounding tissues were collected and examined by gross inspection, biomechanical testing, and histological examination.

**Results:**

A 5–10 nm poly(lactic-co-glycolic acid) (PLGA) film was evenly distributed on MWNTs. The 3% MWNT composite group showed lower cytotoxicity and appropriate release performance, and it was thus tested *in vivo*. In rats with the 3% composite implanted, host cells were prevented from migrating to the ADM at 2 weeks, vascularization was established more rapidly at 12 weeks, and the values for both the maximum load and the elastic modulus were significantly lower than in the ADM-alone group (p<0.01). Histological staining revealed that the MWNT was still not completely eliminated 12 weeks after implantation.

**Conclusion:**

MWNTs were able to carry VEGF to cells or tissues, and the 3% MWNT composite material showed lower cytotoxicity and had an appropriate release performance, which prompted faster vascularization of the ADM than other scaffolds. Nevertheless, the MWNTs induced harmful effects that should be carefully considered in biomedical studies.

## Introduction

The treatment of hernias, especially for those that are large incisional and recurrent, poses a formidable challenge to the abdominal surgeon. Many different methods have been explored to repair these defects, but using non-absorbable materials to achieve tension-free closure of abdominal wall defects is still the most important reconstructive technique. However, these materials may not only cause infection and chronic pain in the surgical area [1], [2], but may also contribute to the dysfunction of other organs, and cause complications such as bowel adherence, obstructions, and fistula formation [3]–[5]. In treating abdominal wall defects, the trend is shifting towards the use of composite materials, which combine the advantages of various individual materials. Biological materials exhibiting good biocompatibility and anti-infective properties are widely used for repairing these types of tissue defects [6], [7]. Acellular dermal matrix (ADM), as a fascial graph replacement, provides a potential alternative for closing hernias and reducing the rate of complications such as adhesions, fistula formation, seromas, and notably, wound infections. Notably, infections are associated with clean-contaminated and contaminated-dirty surgical fields, for which non-absorbable materials are often contraindicated [8], [9]. In addition, ADM shows better mechanical properties, which are useful in reducing hernia recurrence rates [10], [11]. However, studies have shown that ADM has a lower ability than small intestinal submucosa (SIS) to regenerate blood vessels [12], [13].

With novel structural, electronic, and mechanical properties, multi-walled carbon nanotubes (MWNTs) are an important form of carbon that is finding applications in many fields [14]–[17]. The large specific surface area and the interior cavity of MWNTs are expected to play a vital role in transporting various substances to cells and tissues, such as bioactive peptides, proteins, nucleic acids, and drugs [18].

Polyglycolic acid (PGA) is a hydrophilic, highly crystalline polymer with a relatively fast degradation rate. Although structurally very similar to PGA, polylactic acid (PLA) has different chemical, physical and mechanical and properties because of a pendant methyl group on the alpha carbon [19]. Poly (lactic-co-glycolic acid) (PLGA) is a biocompatible and biodegradable linear co-polymer, which can be prepared with different ratios of its constituent monomers lactic and glycolic acid. The most frequently PLGA ratio is 50∶50 (50% lactic acid and 50% glycolic acid). PLGA has been used to meet a wide variety of clinical requirements, such as the fabrication of scaffolds, coatings, fibers, and nanospheres [20]–[23]. PLGA can degrade into the metabolic end-products lactic and glycolic acid, so it has been considered in many studies as a suitable material by biodegradable microspheres, coatings, or encapsulation [24]–[26].

A number of signal transduction systems are involved in vasculogenesis, including vascular endothelial growth factors (VEGFs) and their receptors (VEGFRs), angiopoietin/Tie receptors, platelet-derived growth factors (PDGFs) and their receptors (PDGFRs) and EphrinB2/EphB4 [27]–[29]. Among these, VEGF and its receptors are particularly important. VEGF ligand binds transmembrane receptors, thereby activating intracellular signal transduction receptor molecules [30], [31].

Plasma polymerization, a novel method for surface functionalization of nanotubes and nanoparticles, was used to uniformly deposit ultra-thin films of functional groups on these substance [32]. The main principle of plasma polymerization is that the ionized and excited monomer molecules created by the electrical field bombard and react on the surface of the substrate. These activated molecules may etch, sputter, or deposit on the substrate surface. Due to these characteristics, the plasma technique can be used for polymer surface functionalization of various nanotubes and nanoparticles [33].

Considering the stronger mechanical properties of ADM, its lower vascular infiltration capabilities, and the greater ability to transport MWNTs, we applied composite scaffolding that combined ADM, MWNTs, and VEGF to repair abdominal wall defects in a rat model. We hypothesized that the composite scaffold would be suitable for repairing abdominal wall defects, with a lower rate of complications than the ADM-alone control scaffold.

## Materials and Methods

### Purification and assessment of MWNTs

We treated MWNTs (Wako, Japan) with the following protocol. First, 2g of MWNTs were refluxed in 50 ml sodium hydroxide (2M) at 60°C for 2 h. After cooling, the MWNT suspension was diluted and washed with deionized water until the pH was 6–7, then dried at room temperature. The MWNTs were then calcinated for 30 min at 530°C, suspended in mixed concentrated nitric and sulfuric acid (1∶3 volume ratio), and sonicated for 4 h at 40°C. After washing with deionized water and drying under vacuum at room temperature, the powder was collected and stored at 4°C.

The purified MWNT powder was characterized by X-ray diffraction (XRD, Rigaku, Japan) with Cu Ka (λ = 0.15418 nm) incident radiation from 2 θ = 20° to 80°. The scan rate was 6°/min, with a step size of 0.02°. Fourier transform infrared (FT-IR) spectra were recorded with an EQUINOX 55 spectrometer (Bruker, Germany), operated from 500 to 4000 cm^−1^ at room temperature. Spectra were obtained using a resolution of 4 cm^−1^, and were averaged over 16 scans. Before IR measurement, MWNTs were ground thoroughly with potassium bromide (KBr) and the powder was subsequently pressed into a transparent pellet.

### Modification of MWNTs

Before modification, MWNTs were pre-treated. An MWNT suspension was prepared by mixing 0.1 g MWNTs and 20 µg VEGF with 5 ml deionized water, and sonicating for 2 h at room temperature. After pre-freezing at −80°C for 12 h and vacuum drying at −60°C for 12 h, the resulting powder was stored at 4°C. The MWNTs loaded with VEGF were then modified by a plasma polymerization treatment that deposited PLGA ultrathin films on their surfaces [34]. Briefly, the VEGF-loaded MWNTs were set at the bottom of the plasma reactor, mixed with lactic acid and glycolic acid, and completely dissolved by sonication in the flask, which was connected to the top of plasma reactor. The initial pressure was reduced to less than 2 Pa, and the argon and monomers (lactic acid or glycolic acid) were then introduced into the plasma reactor during the plasma polymerization. The system pressure was measured with a pressure gauge and maintained at approximately 2×10^6^ Pa. The voltage was 50 V and the current was 150 mA. The plasma treatment time was 12 h and the powder was stirred every 2 h with a magnetic bar.

### Composite scaffold fabrication and assessment *in vivo*

The VEGF-loaded MWNT powder was mixed with crosslinked ADM (Jiang Su Dongci Biomedical Science and Technology Co., Ltd, China) with different proportions of MWNTs (w/w: 1%, 3%, 5%, and 7%) to form composite scaffolds, which were measured through histological, SEM, CCK-8, and ELISA methods.

#### 1. Histological and scanning electron microscopy (SEM) examination

The ADM was histologically examined to determine whether there were any cellular components present. The scaffold was fixed in 4% paraformaldehyde for 24 h. After paraffin-embedded tissue blocks were created, the scaffold was sectioned to a thickness of 6 mm for hematoxylin and eosin staining.

To assess the surface morphology of the composite scaffold, the samples were analyzed using a scanning electron microscope (SEM, Philips-XL-30). Briefly, the scaffold was pre-fixed with 2% glutaraldehyde for 2 h at 4°C, washed twice with PBS, and post-fixed in 1% osmic acid for 2 h at 4°C. After two washes with PBS, the samples were dehydrated with an ethanol gradient and dried to a critical point (HCP-2, Hitachi). The samples were then mounted, sputter-coated with gold (BAL-TEC, Philips), and examined with SEM.

#### 2. Cytotoxicity and endotheliocyte proliferation

The CCK-8 assay (Dojindo, Japan) was used to measure the cytotoxicity of composite scaffolds of varying MWNT concentration (1%, 3%, 5%, and 7%) at different time-points (1, 3, 5, and 7 days). An identical procedure was used for each time point. The composite scaffolds (2 cm×2 cm) were immersed in 20 ml Dulbecco's modified Eagle medium (DMEM) containing 10% fetal bovine serum (FBS), and shaken in a shaking incubator for 12 h. The release medium was then collected. Fibroblasts were plated in quadruplicate at a density of 2000 cells/well in 100 µl DMEM containing 10% FBS. After 24 h of culturing in a 5% CO_2_ incubator at 37°C, the cell culture medium was replaced with the release medium. After a further 24 h of culturing, 10 µl CCK-8 solution was added to each well and the 96-well plate was incubated for an additional 4 h. The absorbance was measured at 450 nm using a microplate reader

To assess the proliferative effect of VEGF on endothelial cells, cell viability was tested using CCK-8 assays, as above, after 1, 3, 5, and 7 days of culture, using endothelial cells (4000 cells/well). Endothelial cells (ECs) were selected because cell migration, survival, proliferation and differentiation can be regulated by VEGF in these cells [35], [36].

#### 3. Determining the VEGF content of the scaffold

The VEGF content in the composite scaffolds was quantified with ELISAs. Briefly, 2 cm×2 cm composite scaffolds were immersed in 10 ml PBS in a shaking incubator for 7 days. 0.5 ml of release solution was obtained every day, and stored at 4°C for ELISA analysis according to the manufacturer's instructions. Each sample was run in triplicate and absorbance was measured at 450 nm using a microplate reader (Themo LabSystems, France).

### Experiment design and surgical procedures

For the *in vivo* study, 43 Sprague-Dawley rats (150–200 g) were used. All experiments were approved by the institutional review committee of the Shanghai Jiao Tong University School of Medicine. The animals were divided into three groups. The experimental group (n = 20) received a composite scaffold (ADM, MWNTs, VEGF). The scaffold control group (n = 20) received an ADM-only scaffold. The negative control group (n = 3) had their abdominal wall defects left unrepaired. All animals were sacrificed 2, 4, 8, or 12 weeks post-surgery (five animals at each time-point) for gross inspection, histological examination, biomechanical tests, and SEM. The negative control group (n = 3) was sacrificed at 12 weeks for gross observation of the herniation.

To create the abdominal wall defects, rats were anesthetized by intraperitoneal injections of 10% chloral hydrate (4 ml/kg). The abdominal wall was shaved, disinfected, and covered with sterile draping. A full-thickness abdominal wall defect (3 cm×4 cm), except for the peritoneum (sublay), was created. The edge of the defect was sutured directly to the implanted scaffold with 5–0 silk sutures, and the skin was closed in layers with 3–0 silk sutures.

### Gross observation

After surgery, animals were observed every week for local or systemic complications, including death, hernia, dehiscence, seroma, and infection. When the samples were obtained, they were inspected for vascularization and fibrotic adhesions to surrounding tissues. We graded the adhesions on a numerical score from 0 to 4, according to the following criteria: 0, no adhesions; 1, thin and filmy adhesions easily separable by blunt dissection; 2, definite localized adhesions; 3, definite multiple visceral adhesions; and 4, dense adhesions extending to the abdominal wall [37].

### Histology and immunohistochemistry

To harvest the composite scaffold and the surrounding tissues, rats were sacrificed by high-dose chloral hydrate 2, 4, 8, or 12 weeks after surgery. Tissue samples were fixed with 4% paraformaldehyde, paraffin-embedded, and sectioned to a 6 mm thickness for hematoxylin and eosin staining to examine tissue structure, particularly cell density and neo-blood vessels. In addition, to assess neovascularization and collagen at the repaired site, the samples were analyzed using immunohistochemistry for Von Willebrand Factor antibody (VWF) and collagen I (Col-I) in rats receiving a composite scaffold. Slides were treated with 0.3% hydrogen peroxide for 10 min to block endogenous peroxidase, blocked with 2% bovine serum albumin, and then incubated with rabbit anti-rat monoclonal antibody VWF (1∶4000 dilution, ab6994, Abcam) or Col-I (1∶1000, ab90395; Abcam) for 2 h at 37°C. After three washes with PBS, slides were incubated with goat-rabbit IgG conjugated with horseradish peroxidase for 1 h, then developed with Liquid DAB Substrate Chromogen System.

### Biomechanical testing

Rat tissue samples (1 cm×3 cm) obtained at 1, 2, and 3 months post-surgery were mechanically tested using a biomechanical analyzer (Instron). The length of the tested scaffold was set to 1 cm between the two grippers, which were gradually moved at a speed of 25 mm/min until the complete rupture of the scaffold, to obtain the maximal load and elastic modulus.

### Statistical analysis

All results are presented as mean ± standard deviation. The differences in elastic modulus and maximal load were analyzed using the one-way ANOVA test. A *P* value less than 0.05 was considered statistically significant. The SPSS 16.0 software was used for statistical analyses.

## Results

### Characterization of MWNTs by X-ray diffraction and FT-IR

**Figure 1:**
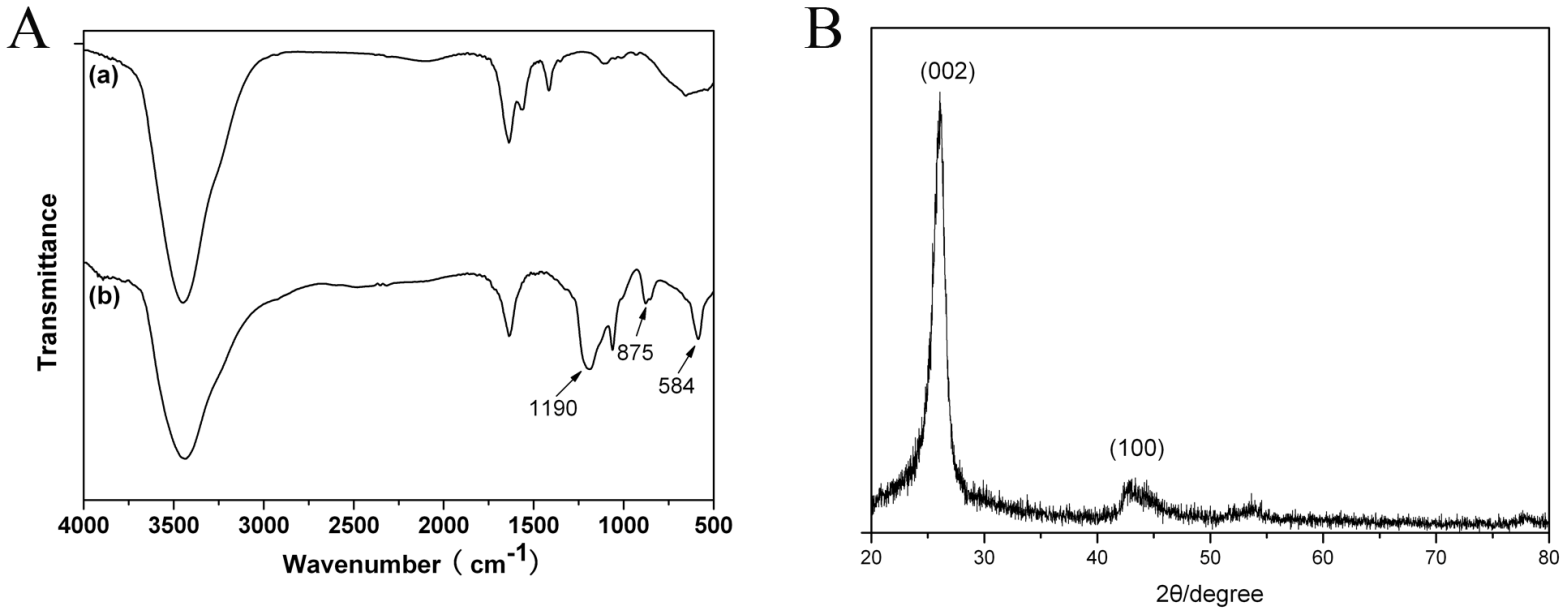
FTIR spectrum (A) and X-ray diffractograms (B) of MWNTs. (a) The FTIR spectrum of MWNTs before oxidation. (b) The FTIR spectrum of MWNTs after oxidation.

The MWNTs were characterized by powder X-ray diffraction (XRD). The diffraction pattern shows major peaks around 2θ values of 26.06 and 43.36, corresponding to the characteristic peaks of (002) and (100) of MWNTs, respectively (Fig. 1B). No other crystalline peak was observed, except for the diffraction peaks attributed to the MWNTs. Unmodified MWNTs only show peaks of C = O (around 1500–1750 cm^−1^), which may arise from defects on the tube walls from the synthesis or purification process. However, the modified MWNTs showed absorption bands at approximately 1000–1300 cm^−1^, 800–1000 cm^−1^, and 600 cm^−1^ after oxidation (Fig. 1A).

### Generation of MWNT-VEGF by plasma modification

**Figure 2:**
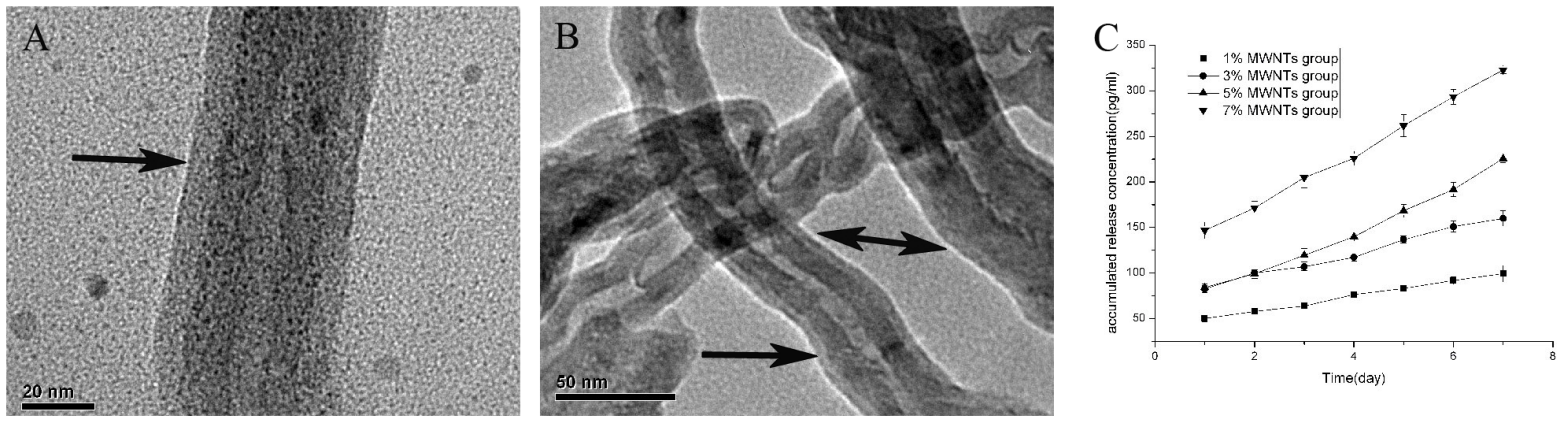
SEM images of MWNTs after plasma polymerization (A, B) and the cumulative release of VEGF (C). (A) Scale bar = 20 µm; (B) scale bar = 50 µm; black arrow indicates the PLGA film.

SEM revealed 5–10 nm PLGA films that were evenly distributed on the MWNTs and caused a sustained-release of VEGF. One or many MWNTs could be coated by PLGA by plasma polymerization (Fig. 2 A, B). The VEGF ELISA kit was used to quantify the release of VEGF from MWNTs during the PLGA degradation process (Fig. 2C). There was no obvious burst release over 7 days. The longer the *in vitro* incubation time, or the higher the MWNT content (w/w), the more VEGF was released from the MWNTs. Over a 7-day period, the cumulative release of the 7% group was 323.03±4.03 pg/ml. Therefore, the PLGA thin film can be considered a sustained-release media.

### Histology and SEM of ADM *in vitro*

**Figure 3:**
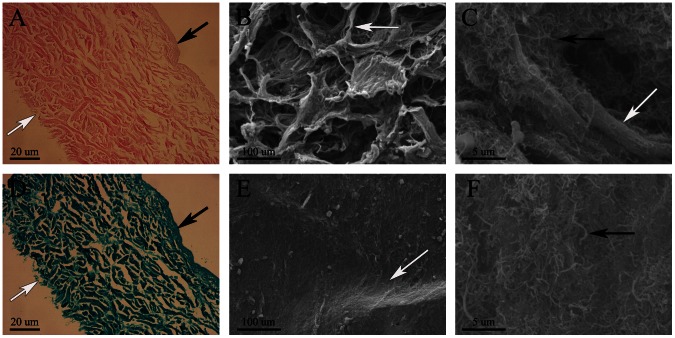
Histological examination (A, D) and SEM images of the surface morphology (B, C, E, F) of the ADM. (A, D) Histological examination of the ADM. (B, C) The surface morphology close to the serosa layer. (E, F) The surface morphology close to the mucosal layer. The black arrow in (A) and (D) indicates the mucosal layer and the white arrow indicates the serosa layer. The black arrow in (B–C) and (E–F) indicates MWNTs and the white indicates the ADM. Original magnifications: (A, D) 100×; (B, E) 500×, scale bar = 100 µm; (C, F) 10000×, scale bar = 5 µm.

H&E staining revealed that no visible cellular material was evident in the ADM scaffold (Fig. 3A, D), and Masson's trichrome staining detected abundant longitudinally-aligned collagen fibers (Fig. 3D). SEM examination showed that the MWNTs mostly adhered to the surface or inside the surface pores of the ADM (Fig. 3C, F). In addition, there were significant differences in surface morphology between the two sides. Close to the serosa layer, pores were much more abundant than on the corresponding opposite side (Fig. 3 B, E).

### Cytotoxicity and proliferation of composite scaffolds *in vitro*

**Figure 4:**
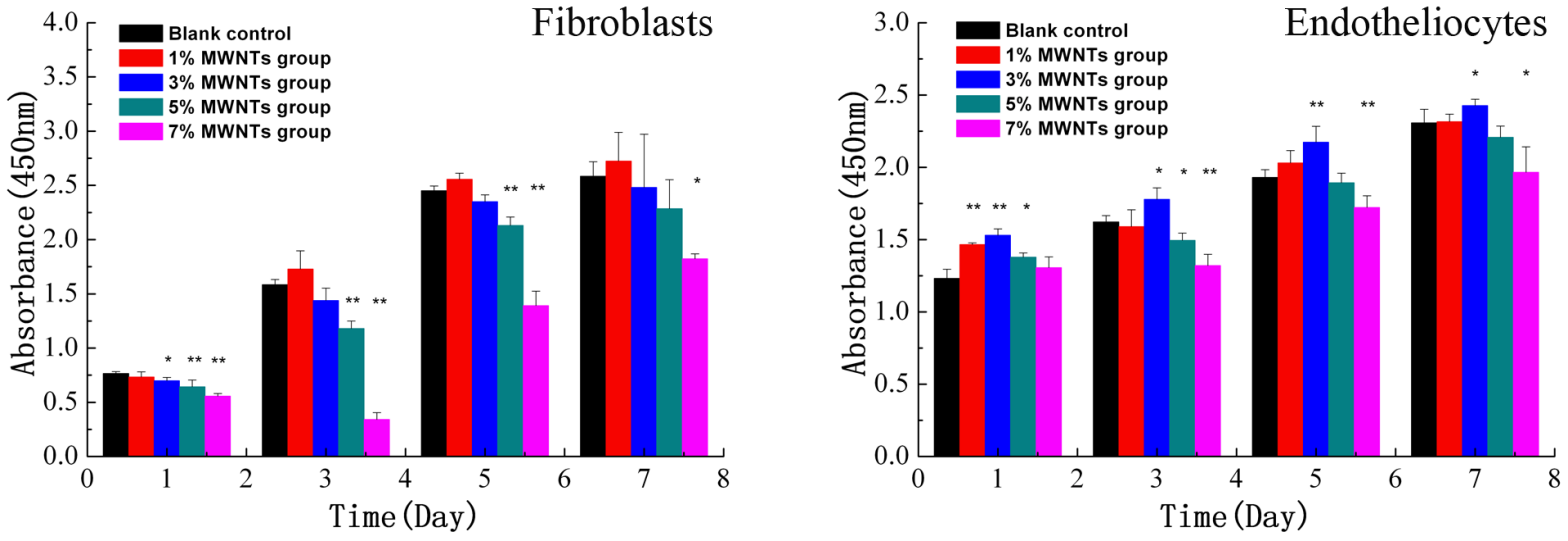
Fibroblast cytotoxicity and endotheliocyte proliferation of CCK-8. * *P*<0.05, **P<0.01.

To further study the biosafety of the composite scaffolds, cytotoxicity was measured by the CCK-8 assay. With increasing concentrations of MWNTs, both fibroblasts and vascular endothelial cells showed slower growth rates (Fig. 4). However, the 1% and 3% MWNT composite scaffolds were associated with better growth than the scaffolds with higher proportions of MWNT. Significant differences were observed between the 5% and 7% MWNTs composite scaffolds and the negative control group, indicating that they caused a slower growth rate.

The proliferative effect on vascular endothelial cells was also monitored (Fig. 4). The cells with the 3% MWNT composite exhibited higher growth.

### Gross observation

**Figure 5:**
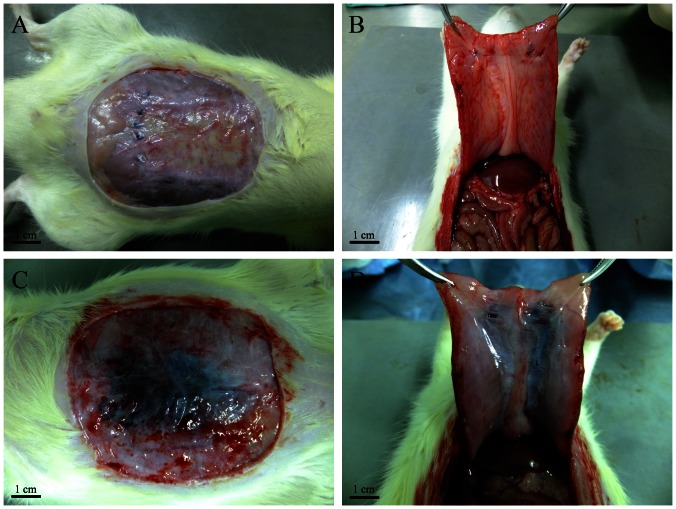
The process of scaffold removal. (A, B) Gross view of the ADM scaffolds 12 weeks after implantation. (C, D) Gross view of composite scaffolds 12 weeks after implantation. (B, D) Gross view of adhesions.

In this study, all rats implanted with scaffolds survived surgery, without signs of herniation after 7 days. However, 6 rats in the experimental group developed seroma within approximately a week, and 4 of them died during the following three days. No infections or adhesions were observed at the repaired sites (Fig. 5 B, D), likely because we preserved the peritoneum, which prevented MWNTs from entering the abdominal cavity. The scaffolds were integrated with surrounding tissue and showed different degrees of degradation. In addition, the surrounding tissue gradually grew to the center of the scaffold.

### Histology and immunohistochemistry

**Figure 6:**
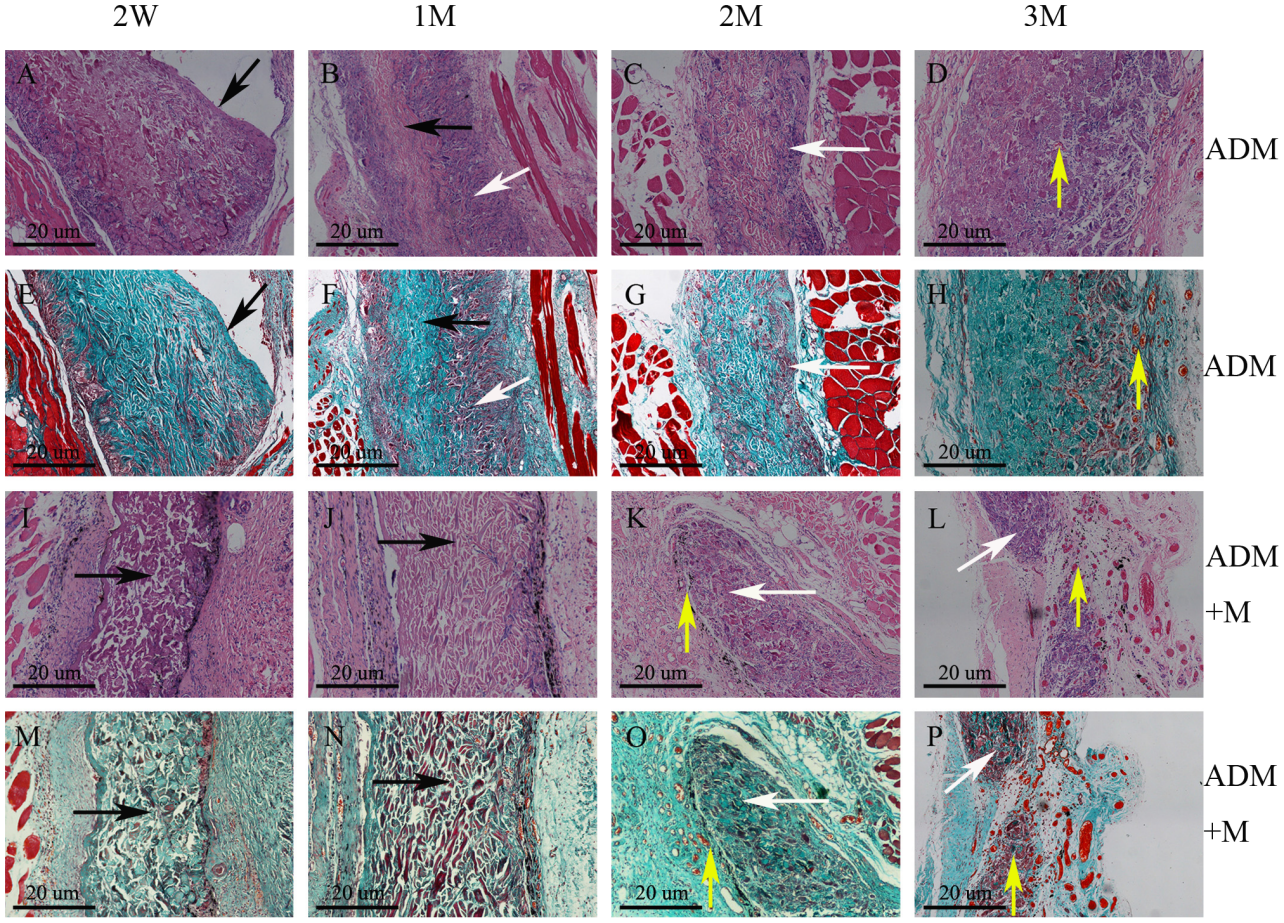
Histological images of the composite scaffolds and ADM constructs 2, 4, 8 and 12 weeks after implantation. (A, B, C, D, I, J, K, L) H&E staining. (E, F, G, H, M, N, O, P) Masson trichrome staining. The black arrows indicate ADM without the host cell infiltration, the white arrows indicate ADM with the host cell infiltration, and the yellow arrows indicate revascularization. Original magnification: 100×. Scale bar = 20 µm.

**Figure 7:**
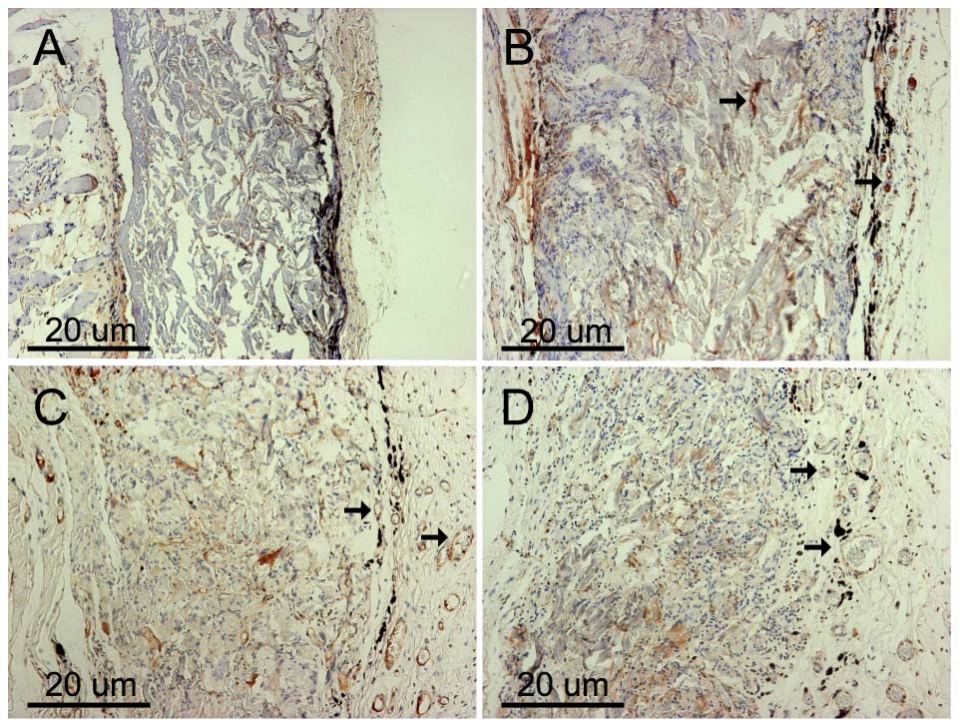
Immunohistochemical staining (VWF) of the composite scaffolds at 2 (A), 4 (B), 8 (C), and 12 (D) weeks after implantation. The black arrows indicate positive VWF. Original magnification: 100×. Scale bar = 20 µm.

**Figure 8:**
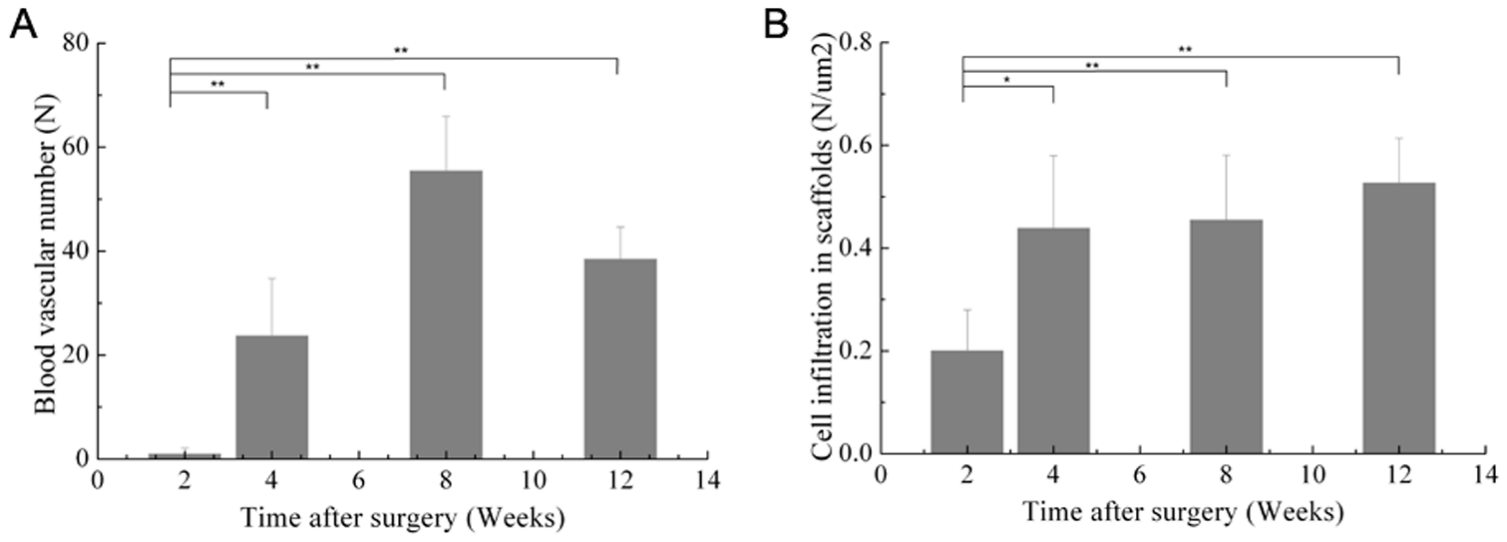
Blood vascular number and cell infiltration in composite scaffolds at 2, 4, 8, and 12 weeks after surgery. * *P*<0.05, **P<0.01.

**Figure 9:**
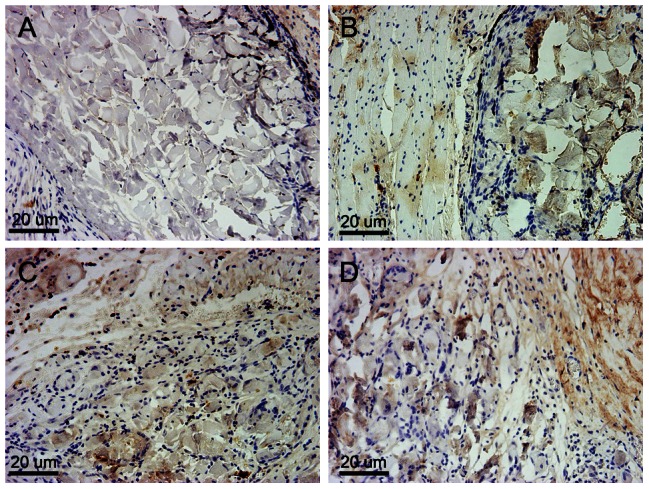
Immunohistochemical staining (Col-I) of the composite scaffolds at 2 (A), 4 (B), 8 (C), and 12 (D) weeks after implantation. The black arrows indicate the ADM and the small black arrow indicates positive collagen I. Original magnification: 200×. Scale bar = 20 µm.

To examine cellular infiltration, ADM degradation, and vessel formation, rats were sacrificed at 2, 4, 8, and 12 weeks for H&E and Masson trichrome staining. H&E staining revealed that all scaffolds in the experimental and ADM-only groups had very few infiltrating cells, which invaded approximately one-quarter to one-third of the thickness of the whole scaffold at 2 weeks. Twelve weeks post-surgery, the host cells could be seen distributed throughout most of the scaffolds (Fig. 6 A, D). However, a significant difference was found between the two sides of the ADM, and some areas close to the mucosal layer had no cellular infiltration. Masson trichrome staining clearly revealed the distribution and degradation of collagen fibers in the ADM. Most of the collagen fibers were completely destroyed with the process of cellular infiltration, and some areas were completely ruptured at 12 weeks (Fig. 6 L, P). The experimental group showed more accentuated degradation of the collagen fibers than the scaffold control group, mostly because there was greater vessel formation. Compared to the ADM-only scaffolds, high density angiogenesis was observed around the experimental scaffolds, notably around the MWNTs (Fig. 6 K, O, L, P). At 12 weeks, an increased number of larger vessels was found in the experimental group (Fig. 6 L, P). These findings were further confirmed by the results of immunohistochemical staining for VWF (Fig. 7). There were fewer blood vessels and less cell infiltration in composite scaffolds at 2 weeks (P<0.01) and high-density angiogenesis was observed in the composite scaffold at 2 and 3 months after surgery (P<0.01; Fig. 8). Immunohistochemical staining for COL-I in composite scaffolds showed that little new collagen was observed at 2 weeks, with more developing 4 weeks after implantation (Fig. 9). In addition, most of the ADM was not obviously stained.

### Mechanical properties

**Figure 10:**
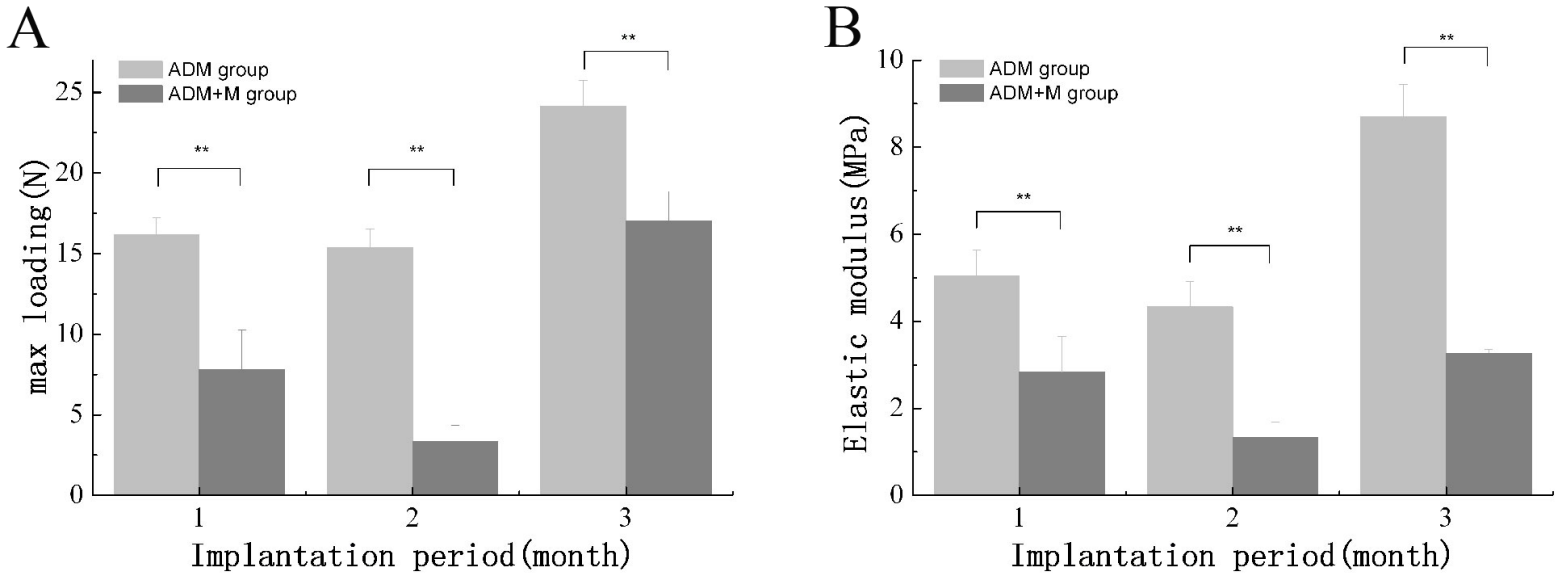
The maximum load and elastic modulus were used to evaluate mechanical properties at 4, 8, and 12 weeks post-surgery. Significant differences were seen in the maximum load and elastic modulus (P<0.01) between the two groups at each time-point. * P<0.05, **P<0.01.

A biomechanical analysis instrument was used to examine the tensile strength of the *in vivo* composite scaffolds. After 4, 8, and 12 weeks of culture, the maximum load values in the composite scaffold group were 7.82±2.45, 3.33±1.02, and 17.04±1.82 N, respectively. However, the ADM-only group had maximum load values of 16.09±1.09, 15.39±1.34, and 24.13±1.62 N, respectively. A significant difference in the maximum load was observed at different time points in both groups (Fig. 10A). Additionally, the elastic modulus of the ADM group was also significantly higher than that of the experimental group (Fig. 10B).

## Discussion

To achieve a tension-free closure of the abdominal wall defects, synthetic materials are frequently used to replace missing fascial tissues and strengthen the repair. Some of these materials may cause local complications, such as infections, chronic pain, erosions, and fistula formation, particularly when they are placed directly over the viscera. While other materials exist, each has its own advantages and complications [8], [38], [39]. Therefore, composite materials, which combine the advantages of their multiple components, may be more effective for treating abdominal wall defects. In the present study, we explored the safety and feasibility of a composite scaffold that contained ADM, MWNTs, and VEGF.

Biological materials have been developed for implantation into abdominal wall defects, particularly SIS and ADM, a three-dimensional tissue implant that contains collagen fibers, elastin filaments, hyaluronan, and proteoglycans. In 2003, ADM was introduced as an alternative to synthetic materials, with suggestions that it has an improved capacity to integrate with surrounding tissues and is less prone than synthetic materials to infection, erosion, extrusion, adhesion formation, and rejection [40]. After implantation into the body, ADM is gradually remodeled into the host tissue and revascularized. While its structural integrity is preserved, it cannot be distinguished from the surrounding tissues. Therefore, it could be widely used to treat abdominal wall defects, particularly for complex situations or when contamination is present [8], [9], [41]. Our previous studies have shown that, for ADM, the mechanical strength is higher, but the effect on vascularization is lower than for SIS [12]. For ADM, in order to ensure rapid angiogenesis, we used MWNTs as a VEGF carrier to promote cellular infiltration and ADM degradation.

MWNTs with a high aspect ratio, high mechanical strength, superior electrical conductivity, and a large surface area, have applications in various fields, including nanoelectronics, nanocomposites, and biomedical disciplines [42]. This material can play a vital role in transporting molecules to cells and enhancing their translocation efficiency [18], [43]. MWNTs have been chosen as a carrier mostly because of their larger inner wall diameters (70–150 nm), which are particularly suitable for delivering molecules of comparable sizes [44]. However, it is difficult to manipulate MWNTs because of their low solubility. Nevertheless, solubilization may be achieved by functionalizing the MWNT surface, via covalent and noncovalent methods [45]. Oxidation with strong oxidizing agents adds hydroxyl and epoxide groups, in addition to the carbonyl and carboxyl groups located at the edges. This enhances biocompatibility and has become a widely-used approach for biomedical applications. In our study, purified MWNTs were oxidized in a 3∶1 mixture of concentrated H_2_SO_4_∶HNO_3_ and sonicated for 4 h to cut MWNTs into 100–500 nm fragments [46]. The FT-IR results showed that more groups were found after the oxidation process (Fig. 1A).

The important parameters for determining the cytotoxicity of MWNTs are their size, surface area, surface chemistry, tendency to aggregate, and impurities [47], [48]. MWNTs that are currently being produced contain a substantial amount of other components, including metal catalysts, graphite, amorphous carbon, and carbon nanoparticles. Some metal catalysts have been reported to have toxic effects. Cobalt and nickel cause cytotoxic or genotoxic effects as well as lung diseases such as fibrosis, interstitial lung disease, and asthma [49]. The efforts to remove impurities can generate some functional groups or defects, which influence cytotoxicity [42]. Toxicity of MWNTs is still a controversial topic. Some studies have reported harmful effects, while others have observed either no toxicity or even found beneficial effects [50]. The potential toxicity of the composite scaffold mainly comes from the MWNTs and, therefore, it is necessary to detect toxicity before using these scaffolds *in vivo*. Increased levels of MWNTs inhibited fibroblast and endothelial cell growth, and the CCK-8 results of the 5% and 7% MWNTs groups indicated lower than normal levels of cell growth, especially for the 7% composite. This demonstrates that MWNT cytotoxicity is dose-dependent (Fig. 4). The 3% MWNT composite group showed normal or slightly higher values than those in the normal group for both cell types. In the present study, implantation of the 3% MWNT composite did have significant effects on cell growth *in vitro*, but there were some regions of inflammation and granuloma *in vivo*. Meanwhile, *in vitro* experiments were able to achieve a better dispersion of MWNTs, but in *in vivo* experiments, the MWNTs could still aggregate, which could increase local cytotoxicity [50], [51].

The effective repair of abdominal wall defects depends on the early re-establishment of angiogenesis and cellular infiltration [52]. Early revascularization could lead to more host cells entering the ADM, which accelerates its degradation and ensures better defect repair. This study aimed to develop a PLGA film around MWNTs by a plasma polymerization treatment that could effectively load and sustainably release VEGF. The PLGA film can degrade into the metabolic end-products lactic and glycolic acid. Both acids are innocuous, since they are incorporated into the Krebs cycle and excreted in the form of carbon dioxide and water. With the degradation of the PLGA films, VEGF is released to promote angiogenesis. It is reported that VEGF ligand binds and activates transmembrane receptors, leading to initiation of intracellular signal transduction receptor molecules and regulates vasculogenesis [30], [31]. While not all MWNTs can be wrapped into PLGA films, VEGF can still be adsorbed by MWNTs and then released. The current study demonstrates that the sustained-release effect gradually increases with time; it does not occur as a burst release at a specific time point. Comparing the experimental results above (cytotoxicity and sustained-release effect), we chose the 3% MWNTs composite group, which exhibited lower cytotoxicity and appropriate release performance. In our study, histological analyses revealed that it took host cells 3 months to infiltrate the whole ADM, and few small blood vessels could be found surrounding the ADM. However, in the composite group, the same infiltrationcould be achieved at 1 month (Fig. 6 J, N): a high density of blood vessels was found surrounding the MWNTs, and some were in the ADM at 2 months (Fig. 6 K, O). Some regions of the ADM were completely degraded because of rapid vascularization (Fig. 6 L, P). In addition, in both groups, and particularly in the composite group, at 2, 4, and 8 weeks, host cell infiltration reached one-quarter to one-third, one-half to two-thirds, and the full thickness of ADM, respectively. It is worth mentioning that there are significant differences in the surface morphology between the two sides (Fig. 6). Close to the mucosa layer, cell infiltration is less than at the corresponding opposite side, which explains why portions of the ADM were still without cellular infiltration.

Mechanical properties play an important role in reconstructing abdominal wall defects. The process of reconstruction can be divided into two parts: the presence of collagen fibrils of the ADM before degradation and the formation of neo-tissue after implantation. If the scaffold has sufficient mechanical strength to prevent the occurrence of hernia before its complete degradation, the hernia recurrence rate would be significantly reduced. In the present study, both the maximum load and the elastic modulus were significantly higher in the AMD-only group than in the experimental group (Fig. 7), mostly because the rapid vascularization led to the degradation of the ADM, the main structure ensuring early mechanical strength. Even at the lowest mechanical strength (3.33±1.02), no herniation was observed in the experimental group. Another possible factor is the collagen hydration enhanced by MWNTs absorbance. The effect of hydration is of great importance. Meyers has shown that the calculated density of collagen decreases from 1.34 to 1.19 g/cm^3^ with hydration and is accompanied by a decrease in the Young's modulus from 3.26 to 0.6 Gpa [53].

One limitation of the present study should be mentioned. We only assessed the short-term effects of the composite scaffold, and long-term studies are needed to evaluate its toxicity and mechanical properties.

## Conclusions

A composite scaffold has been generated with ADM and VEGF-loaded MWNTs by a modified plasma polymerization method. The 3% MWNT composite scaffold is less cytotoxic and shows appropriate release performance. Controlled release of VEGF by the plasma polymerization treatment offers a method to accelerate early revascularization, and the 3% MWNT composite scaffold was able to repair abdominal wall defects. MWNTs can be an efficient molecular transport system because of their high loading efficiency and other features. However, the cytotoxicity associated with MWNTs must be carefully considered when employing them in biomedical applications.
